# Simulation‐based education: A narrative review of the use of VERT in radiation therapy education

**DOI:** 10.1002/jmrs.276

**Published:** 2018-04-14

**Authors:** Paul Kane

**Affiliations:** ^1^ Department of Radiation Therapy University of Otago Wellington New Zealand

**Keywords:** Education, radiation therapy, simulation, VERT

## Abstract

Simulation has a long history in medical and health science training and education. The literature describing this history is extensive. The role simulation plays in many health disciplines has evolved, as has the focus of the literature around it. The Virtual Environment for Radiotherapy Training (VERT) system is a relative newcomer to radiation therapy education and, similar to the literature around radiation therapy (RT) education, is still in its infancy. This narrative review sets the scene of simulation‐based education within the health sciences and considers the lessons learned from published work on VERT to date. The evidence suggests that future inquiry involving VERT should explore different ways in which VERT can be used to contribute to the skillset required by the radiation therapist of tomorrow.

## What This Article Sets Out to Do

When considering areas of enquiry, it is often useful to perform an appraisal of the ‘state of play’ of existing literature. Systematic reviews, for example are a rigorous way in which current literature can be appraised. The chief limitation of such reviews is that a narrow scope is permitted by the fairly rigid constraints set by the strict guidelines for inclusion criteria of articles of interest. A useful alternative is the narrative review. What they lack in established format is compensated for with an ability to appraise the reader of what literature exists around a topic and what potential there is for further lines of enquiry.^1^ Such reviews do not necessarily involve a detailed critique of each article considered, as the intent is not to provide a basis for clinical decision making, but rather encourage consideration of a broader topic.^2^

The aim of this narrative review is to provide a perspective on enquiry concerning the Virtual Reality Radiotherapy Training or VERT system. This aim is achieved by meeting three objectives: first, establishing a context for the use of simulation in health science education; second, identifying the themes to research already conducted around VERT and finally, identifying potential future directions for research into the VERT system.

Databases such as PubMed, Medline, EBSCO, CINAHL and Google Scholar were used to find articles relating to simulation in health science education. The focus was on systematic review articles as well as editorials and articles providing an overview. Those same databases were used to find any articles relating to the VERT system.

## Background of Simulation in Health Science Education

Like many health professions, there are significant demands placed on the cognitive and motor skills of a radiation therapist. Skills are often described as being acquired rather than learned. The adage ‘repetition is the mother of a skill’ encapsulates the need to provide trainee practitioners opportunities to develop such abilities. Many occupations require frequent practice of skills to achieve mastery, such mastery is often a precursor to being permitted to practise those skills independent of supervision. During those iterations, inevitably the learner will make mistakes, some of them significant. In the past, healthcare disciplines have relied on an apprenticeship model of training where, under supervision, the student will ‘see one, assist with one, do one’.^3^, ^4^ While the apprenticeship model arguably has its place in the health professions, the concept of making mistakes to afford learning opportunities is not acceptable when people and their health and wellbeing are involved.^5^ To enable students to learn patient‐centred skills safely, there is an increasing role for simulation. Bradley offers a useful definition of just what is meant by simulation in the health science context.^6^

The technique of imitating the behaviour of some situation or process …… by means of a suitably analogous situation or apparatus, especially for the purpose of study or personnel training. (page 254)

An extensive literature illustrates the number of healthcare disciplines using simulation‐based teaching and learning in their curricula. Examples are found in surgery, anaesthesia, dental surgery, inter‐professional learning, emergency medicine, diagnostic skills, history taking and trauma medicine.^7^, ^8^, ^9^, ^10^, ^11^, ^12^, ^13^, ^14^

## The Case for Simulation

A study which surveyed educators in various health disciplines found strongly held perceptions that simulation was beneficial to teaching, learning and assessment across a range of domains. These included but were not limited to procedural skills, communication skills, interdisciplinary interactions and patient evaluation skills.^15^ There has been significant reform in medical, nursing and other health discipline education in recent times.^6^, ^16^ This reform is indicative of a move away from the apprenticeship model to one based on social interaction and experience, focused on quality of care for the patient.^17^, ^18^ Cioffi outlined a series of perceived advantages to using simulation; learning is experiential, active, iterative and mimics clinical reality while removing the risks associated with failure.^19^ Such learning approaches can fall into the constructivist school of thought. This model emphasises that a learning environment must be ‘safe’ for the student, permitting opportunities to try, fail and not be subject to derision or ridicule.^18^ Kneebone and Kunkler (amongst others) discuss the ability of simulation‐based learning to provide such an environment for students.^3^, ^8^ Learning experiences based in such an environment are more likely to encourage deep learning and are therefore more likely to be lasting for the student.^20^, ^21^ Moule acknowledged that simulation goes beyond simple demonstration of clinical skills. Rather students may practice, receive feedback and ‘try again’ with the confidence that no harm will come to patients.^22^ Attention to patient safety has arisen in no small part due to the reform which has occurred in medical and other health professional education refocussing on patient needs.^6^ What Bradley terms the ‘safety agenda’ has been brought to the fore in a series of themed publications.^23^, ^24^ Gaba discussed simulation‐based training in anaesthesiology as a model for patient safety in such an issue.^25^ Ziv and colleagues strongly advocated for the use of simulation‐based training to improve patient safety in healthcare delivery^26^ and describe integrating simulation into healthcare training as an ethical imperative.^5^ This article definitively positioned the patient as needing their health issues addressed, not to serve the training needs of the student practitioner.

## Issues with Simulation

In their review of medical simulation history, Rosen has pointed out that medicine is another example of a concerning phenomenon with respect to simulation:

No industry in which human lives depend on the skilled performance of responsible operators has waited for the unequivocal proof of the benefit of simulation before embracing it^17^

There are other examples of reviews or studies where the evidence of benefit from simulation‐based education versus not using simulation is equivocal.^16^, ^17^, ^27^

There is concern that while students can successfully learn clinical skills using simulation techniques, there is a risk that the patient is not fully considered in subsequent clinical practice; in a sense, simulation risks generating a false sense of security. Such limitations can happen irrespective of the simulation being high or low fidelity, how closely the simulation mimics reality. A simulation has limitations and may lack realism, using standardised patients may limit the variety of patient experience which may be encountered.^28^ Such limitations could be characterised as a failure in the transfer of learning. Norman et al.^29^ conducted a review specifically examining this issue. They concluded that studies trialling simulation against no intervention do not clearly show a relationship between how material is taught and what is learnt. This represents a significant challenge and muddies the waters with respect to the benefits of simulation. Cook et al.^12^ reviewed 609 studies and concluded there was no merit in further studies comparing simulation versus no simulation as only 4% of the studies identified did not show any benefit. This group argued a more useful direction would be to examine when and how best to utilise simulation in any teaching curriculum.

Some of the studies discussed above illustrate a major philosophical criticism of the use of simulation based education; a lack of underpinning theory. Cant and Cooper suggested that while evidence existed of derived benefit there is still much to be learned with respect to effective implementation.^16^ Bradley and Postlethwaite identified that a paucity of sound educational research is commonplace with regard to new ideas in healthcare training.^18^ Berragan indicated that successful use of simulation requires awareness of the range of theoretical models of learning; there is no ‘one size fits all’ approach which can be utilised.^28^ Gaba highlighted the lack of high‐level evidence confirming the benefits of simulation, he asserted the appropriate measure was data equivalent to a randomised clinical trial.^30^ Educational studies often depend on self‐reporting from students to measure the success of using simulation.^31^, ^32^, ^33^, ^34^ While this would not appear to be the high‐level evidence Gaba calls for, such studies are appropriate for educational research. Bradley and Postlethwaite highlighted the importance of choosing an appropriate paradigm of inquiry for educational research.^18^

## The Case of VERT

The Virtual Environment Radiotherapy Training (VERT) system is a virtual reality environment presenting the user with a linear accelerator (LINAC), patient couch, the bunker the equipment resides in and a virtual patient. Interaction with the system has varying degrees of immersion but at a minimum involves actual LINAC control handsets from the major equipment vendors interfaced with the software and touch screen controls. This allows control of the virtual equipment. The environment can be presented in two dimensional (2D) or three dimensional (3D) form. Additionally treatment beams can be visually represented and the patient can be presented as a ‘solid’ person or their internal anatomy can be represented by rendering of those organs or related CT images. The two abilities just described can be combined with imported treatment plans to truly represent in 3D the dosimetric impact of a given treatment technique or set up. The fully immersive version of the system allows what the user sees to be controlled by movement of the user around the virtual environment. The chief limitation is the inability to manually position a virtual patient on the treatment couch. Even with this taken into account, VERT provides a relatively high‐fidelity experience for the student practitioner.

The existing literature on VERT as a simulation tool for radiation therapy training is, in contrast to simulation in health science generally, limited. This can be explained in part by the fact that the system only became available commercially in 2008. Additionally, radiation therapy is a relatively small discipline in the context of healthcare. The inventors of the system initially published the development of VERT as a concept and later provided a description of the capabilities and features of the operational system. Some context was outlined as to the potential benefits of the system in the training of radiation therapists (known as therapy radiographers in the UK). Early uptake of the system was primarily intended to address workforce issues in England and allow students the development of motor skills without pressurising clinical resources.^35^, ^36^

Development of the system continued with a series of partners in academic and clinical settings with publications reporting on this progress. Bridge et al.^37^ published findings of a quasi‐experimental evaluation. Their aim was to assess the success of VERT in aiding students to understand skin apposition set ups. There was acknowledgement that simulation lacked the feedback which would be gained in situations with a real patient. However, the ability to practice the task without the burden of a real patient and the inherent constraints that brings was deemed useful by students. From a development point of view the study resulted in some suggestions for additional features which have found their way into newer versions of the system. Green and Appleyard published further work on developing skill with the skin apposition technique.^38^

Three publications of an evaluative nature centred on how the system had been or was going to be used. Two focused on the English context,^39^, ^40^ the third related to the system installed in Aarhus, Denmark.^41^ Appleyard and Coleman wrote a report for the Department of Health who provided funding so that all English education providers and public treatment centres could obtain a VERT installation. The report suggested that VERT applications might be better developed in academic centres.^39^ James and Dumbleton provided further insight into how those installations were used in the clinical context. They reported variation in the use of VERT between centres. Data suggested the full potential of the system was not being realised and this was largely attributable to lack of resources diverted to implement VERT in the respective departments.^40^ Boejen and Grau outlined some of the alternatives for simulation in radiation therapy training but centred their attention on the potential represented by VERT as a more comprehensive approach to radiation therapy simulation. No conclusive data were presented as the paper was more of a discussion in nature.^41^

Among the more recent publications is an article addressing the need for theoretical underpinning of VERT as an educational tool using a specific learning resource as an example. The design and evaluation of the resource were guided by a specific theory of learning and teaching, Constructive Alignment as described by Biggs and Tang.^21^ This is the first time such an approach has been used. This is also the first publication to address the reported under‐utilisation of VERT in the English context.^42^

Motor skill familiarisation has been a hallmark of previously published work. More recently the inventors of the system recently described the ability to demonstrate the dosimetric impact of equipment mis‐calibration in the optional physics module. A good example of expanding VERT use to applied conceptual understanding.^43^

From this brief summation of the literature the reader can ascertain that much remains to be explored about simulation as an educational tool in health science in general and VERT in particular. The existing research on VERT focuses on two main themes; what users foresee the VERT system to be capable of providing and introducing students to basic clinical motor skills. Because of the narrow range in the existing literature around VERT, there are extensive gaps in published understanding of the system and its use as an educational tool. There are a broad range of topics that are worthy of further investigation such as treatment planning concepts, patient education, medical physics and anatomical instruction.

## The New Zealand Experience

The Bachelor of Radiation Therapy (BRT) offered by the Department of Radiation Therapy at the University of Otago, Wellington, New Zealand produces competent practitioners eligible for registration and licencing to practice in the New Zealand health system. The standard of the programme is such that a typical BRT graduate meets the required standard for practice in comparable jurisdictions such as Australia, the United Kingdom, Canada and many mainland European countries. As competence is determined to be the final arbiter of a practitioner's standard of education, it was considered inappropriate that any study would seek to measure or establish if integration of VERT technology would result in ‘better practitioners’. This would imply that those who went before were not up to standard or as competent and that is patently not the case.

A number of studies have been completed or are in progress in our department. An example appears in this issue, reporting on the development and evaluation of a specific VERT teaching module to facilitate first year student understanding of a variety of treatment techniques.^44^ Previously, we have reported on the use of VERT in second year treatment planning academic papers encompassing data collected from students, academic teaching staff, clinical staff and an inventor of the machine.^45^ That study suggested VERT presented a two‐edged sword; on one hand, VERT can facilitate a rethink of the way we educate radiation therapists, on the other, integrating the system into an existing curriculum without thorough preparation and resourcing can provide just as much frustration as benefit. In addition, an interview with the inventor of the system identified that the New Zealand focus on using VERT to support conceptual teaching was more closely aligned to the original intent of the system than the English experience had presented to date. Using VERT as part of an holistic approach to assessing the integration of academic knowledge and soft clinical skills has been reported on.^46^ That study demonstrated students can effectively use VERT to support and demonstrate their conceptual learning. The level of success in this regard however may be linked to the level of training they receive in hands on use of VERT. A broader summation of the variety of ways VERT has been integrated into teaching across the BRT has been published.^47^ A key aspect of that report was the usefulness of VERT in making the longitudinal and horizontal connections between aspects of the entire programme of study. In addition to the student radiation therapist focused studies, in depth work using VERT as a patient education tool to prepare and inform patients prior to a course of radiation therapy has been presented at a number of international forae.^48^

## Future Directions

What the experience in our department has demonstrated is that the growth of active research into the use of simulation and specifically VERT as a teaching and learning tool is mirroring the pathway established by other disciplines. Radiation therapy does not need to explore the question of using simulation or not, rather future research should explore the best ways to utilise the potential benefits as well as avenues not previously thought of. Bridge et al.^49^ have tapped into this thinking with their report on an extended simulation environment of which VERT was a single component. VERT gained a foothold internationally to address workforce issues by providing a tool to permit a different approach to syllabus delivery which freed up precious clinical resources. The New Zealand experience would seem to indicate that moving to integrating technology like VERT at a curriculum design level has potential benefits for teaching staff, students and patients alike. Conceptual learning, opportunities for reflection and critical analysis of multiple clinical scenarios with zero impact on patients’ actual health journeys and exposure to teamwork in a safe environment are all within the scope of simulation and VERT in particular. Educational experience of this nature will provide practitioners of the future with the kind of sustainable lifelong learning our profession needs.

## Conflict of Interest

The author declares no conflict of interest.
